# The role of proactive therapeutic drug monitoring in guiding infliximab therapeutic optimization in pediatric patients with Crohn's disease: A retrospective study

**DOI:** 10.1002/pdi3.96

**Published:** 2024-06-25

**Authors:** Junya Song, Huihui Zhang, Huihua Zhang, Ximing Xu, Xiaohua Liang, Yongfang Liu, Xiaomei Song, Hong Guo, Xue Zhan, Jinlin Song, Xiaoqin Zhou

**Affiliations:** ^1^ Department of Gastroenterology Children's Hospital of Chongqing Medical University (CHCQMU) Chongqing China; ^2^ National Clinical Research Center for Child Health and Disorders Ministry of Education Key Laboratory of Child Development and Disorders Chongqing Key Laboratory of Pediatrics Chongqing Key Laboratory of Child Health and Nutrition Chongqing China; ^3^ Big Data Center for Children's Medical Care Chongqing China; ^4^ Clinical Epidemiology and Biostatistics Department Children’s Hospital of Chongqing Medical University Chongqing China; ^5^ Department of Nutrition Children's Hospital of Chongqing Medical University Chongqing China; ^6^ Department of Gastroenterology Chongqing General Hospital Chongqing China; ^7^ College of Stomatology Chongqing Key Laboratory for Oral Diseases and Biomedical Sciences Chongqing Municipal Key Laboratory of Oral Biomedical Engineering of Higher Education Chongqing China

**Keywords:** Crohn's disease, infliximab, pediatric, therapeutic drug monitoring

## Abstract

Therapeutic drug monitoring (TDM) plays an important role in guiding treatment plan adjustments and clinical outcomes in Crohn's disease. To evaluate the role of TDM‐guided optimization of infliximab dosage in patients with pediatric Crohn's disease. Medical records of patients with pediatric Crohn's disease who were treated with infliximab and had proactive TDM from June 2020 to June 2022 at the Children's Hospital of Chongqing Medical University were included. Baseline influencing factors for infliximab trough concentration (TC) and clinical outcomes before and after the treatment change were analyzed to assess the value of adjusting treatment in the patients. Forty‐six patients (male‐to‐female ratio = 1.55:1, age <18 years) were included. Univariate and multivariate analyses showed that hormone exposure (odds ratio: 0.042, 95% confidence interval: 0.002–0.924, *p* = 0.044), perianal lesions (5.813, 0.984–34.349, *p* = 0.052), simplified endoscopic score for Crohn's disease (1.656, 1.065–2.577, *p* = 0.025), and total protein (TP) (0.851, 0.749–0.967, *p* = 0.014) were correlated with infliximab TC. Shortening the treatment interval increased the infliximab TC (pre vs. post = 1.69 ± 0.8 vs. 12.03 ± 6.64, *p* = 0.001, *n* = 12) after 93.9 ± 37.47 days, decreased the pediatric Crohn's disease activity index and simplified endoscopic score for Crohn's disease, and increased the biochemical remission, clinical remission, endoscopic remission, and endoscopic response rates; however, there was no statistical significance. Hormone exposure, perianal lesions, simplified endoscopic score for Crohn's disease, and TP levels before the first infliximab use affected the infliximab TC. Shortening the treatment interval can improve infliximab TC levels and clinical outcomes.

## Graphical abstract

To understand the clinical guiding value of treatment optimization based on therapeutic drug monitoring (TDM) in pediatric Crohn's disease patients treated with infliximab. Our research finds shortening the treatment interval under the guiding of TDM can improve the infliximab trough concentration level and clinical outcomes.

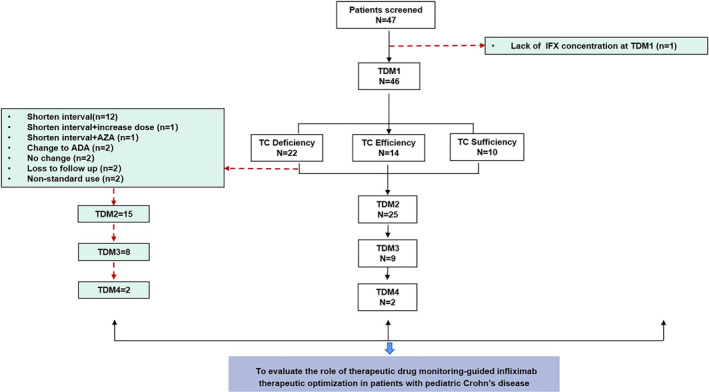

## 1 INTRODUCTION

Inflammatory bowel diseases (IBDs), including Crohn's disease (CD) and ulcerative colitis, are chronic, relapsing, progressive, and disabling conditions that affect the gastrointestinal tract.^1^ Patients with pediatric CD (pCD) account for approximately 15%–25% of all CD patients and usually exhibit a more severe phenotype than adults.^2^, ^3^, ^4^ They are more likely to experience malnutrition, impaired growth, reduced quality of life, and a higher risk of later surgery.^5^, ^6^, ^7^

Infliximab (IFX), an anti‐tumor necrosis factor alpha (TNF‐α) monoclonal antibody, is increasingly being used for pCD. According to European Crohn's and Colitis Organization (ECCO) and Chinese Medical Association guidelines,^8^, ^9^ IFX is used as a first‐line therapy for children with CD, which has improved treatment efficacy, lowered the rate of surgeries, and reduced utilization of other healthcare resources related to complications or worsening of the disease.^10^ Although IFX's clinical response (CRe) rate is high, as much as 10%–30% of the patients do not respond to induction therapy (primary loss of response, pLOR), and approximately 50% of initial responders lose response at a later time (secondary LOR, sLOR).^8^ LOR leads to an increase in disease recurrence, hospitalization, and surgery rates. Thus, optimizing treatment to minimize LOR is of great clinical significance.^11^

Therapeutic drug monitoring (TDM) of serum trough levels of IFX (TLI) and of antidrug antibodies (ADA) provides objective data to evaluate LOR and guide treatment adjustments. The use of reactive TDM according to disease recurrence, recurrent clinical symptoms, and inflammatory index activity is more common than proactive TDM.^12^ However, routine or proactive TDM has greater guiding value than reactive TDM, irrespective of target concentration range, lower risk of ADA, therapeutic outcomes, cost‐effectiveness, or cost‐saving.^13^, ^14^, ^15^, ^16^, ^17^, ^18^ In children, increased disease severity and substantial interpatient variability in pharmacokinetic parameters drive an even greater need for proactive TDM.^8^, ^19^ TDM‐guided IFX dose intensification is also associated with a higher rate of composite sustained greater treatment persistence, corticosteroid‐free clinical remission (CR), normal C‐reactive protein (CRP), and normal fecal calprotectin.^20^, ^21^, ^22^

In this study, we aimed to investigate the use of proactive TDM in pCD with IFX and its use in guiding dose intensification and efficacy evaluation.

## 2 METHODS

### 2.1 Population and research design

This retrospective study included patients from the Department of Gastroenterology at the Children's Hospital of Chongqing Medical University from June 2020 to June 2022. We first analyzed all patients who underwent TDM during the study period and included 47 patients according to the following criteria. The pCD diagnostic criteria were set according to expert consensus and guidelines on the diagnosis and management of pediatric IBD.^8^, ^9^ Proactive TDM was defined as measuring TLI and ADA levels at the end of induction without pLOR or maintenance without sLOR. Reactive TDM is defined as performing TDM at the time of the disease's active stage, pLOR, or sLOR.^23^ The inclusion criteria were hospitalization with a definite diagnosis of CD according to ECCO and Chinese guidelines^8^, ^9^; IFX used as induction and maintenance therapy; IFX induction at 0, 2, and 6 weeks, and maintenance at every 8 weeks, at a dose of 5–10 mg/kg according to weight; and at least one proactive TDM during maintenance. The exclusion criteria were children with undiagnosed CD, non‐standard IFX use, reactive TDM, or no TDM.

The retrospective clinical data of patients with pCD were collected by chart review from electronic medical records. The general demographic data (including age, sex, height, weight, and body mass index [BMI]) and clinical indicators (including the Paris classification of disease diagnosis phenotype,^24^ pCD activity index [PCDAI], simplified endoscopic score for CD [SES‐CD], and biochemical blood indexes) of patients using IFX for the first time were included. Additionally, the clinical data and disease outcome assessments corresponding to each TDM were included. TDM‐guided treatment optimization was defined as treatment adjustment according to the previous TDM results.

### 2.2 Classifications and definitions

Based on the ECCO and China consensus on pCD,^8^, ^9^ PCDAI <10 was classified as remission or quiescent stage, 10–27.5 as mild activity stage, 30–37.5 as moderate stage, and >40 as severe stage.

TDM was performed before subsequent IFX infusion. The IFX concentration was determined using a fluorescence immunochromatography IFX detection kit (Suzhou Herui BioMed Co., Ltd.) at the Suzhou Herui IBD Diagnostic Technology Research Center. Concentrations <3 μg/mL were considered insufficient, 3.1–7 μg/mL as efficient, and >7 μg/mL as sufficient.^25^ An antibody to IFX (ATI) level <30 ng/mL was defined as negative.^26^ The ATI detection kit (Suzhou Herui BioMed Co., Ltd.) is a quantitative fluorescence immunochromatographic assay tool.

Biological remission was defined as CRP ≤8 mg/L and erythrocyte sedimentation rate (ESR) ≤20 mm/h.^27^ CR was defined as PCDAI <10, and PCDAI CRe was defined as a decrease in PCDAI ≥15 from baseline and a total score of ≤30.^28^, ^29^, ^30^ Endoscopic remission (ER) was defined as SES‐CD ≤2, and endoscopic response (ERe) was defined as a reduction of at least 2 points and a reduction of at least 50% in the SES‐CD from the initial score.^31^, ^32^, ^33^

### 2.3 Statistical analyses

All statistical tests were performed using SPSS software version 25.0 (SPSS, Chicago, IL, USA) and GraphPad Prism version 5 (GraphPad Software, San Diego, CA, USA). For quantitative variables, data are shown as mean ± standard deviation (SD) or as median and interquartile range (IQR), according to the presence or absence of a normal distribution. Categorical variables are expressed as percentages. Independent continuous variables were compared using the Student's *t*‐test and Mann–Whitney *U* test, whereas dependent continuous variables were compared using the paired *t*‐test and Wilcoxon signed‐rank test. Categorical variables were compared using the chi‐square test and rank‐sum test. A multivariable binary logistic regression was performed to determine the independent effects of variables associated with IFX trough levels, including statistically significant variables from univariate analysis, based on the backward selection method. *P*‐values <0.05 in the final multivariate model were considered significant.

## 3 RESULTS

### 3.1 Demographic and clinical characteristics

**FIGURE 1:**
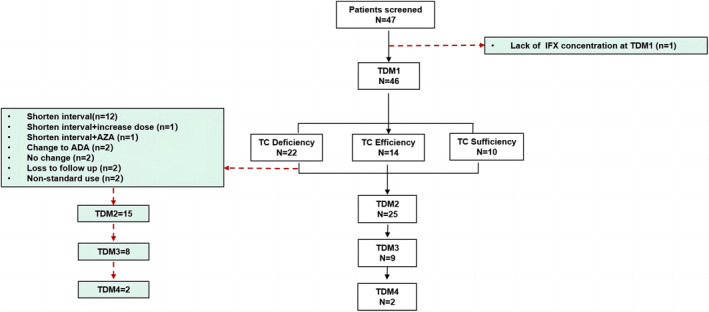
Flow diagram. One patient lacked IFX concentration at TDM1, but had other results at TDM1, including ATI, and also had IFX concentration at TDM2 and TDM3. ADA, adalimumab; IFX, infliximab; TC, trough concentration; TDM, therapeutic drug monitoring.

Patient inclusion and overall experimental design are shown in Figure 1. Forty‐six pediatric patients with CD (average age: 13.42 years, age range: 11–14.44 years) were enrolled, including 28 males and 18 females, with a male‐to‐female ratio of 1.55:1. The patients' height and weight (mean ± SD) were 149.54 ± 14.64 cm and 33.39 ± 9.08 kg, respectively, and their BMI (median (IQR)) was 14.6 (13.3–15.43). The malnutrition rate was 71.7%. When IFX was used for the first time, 67.4% of patients had been hospitalized once, 15.2% twice, and 17.4% three times. None of the 46 patients had ever used biological agents; 54.3% had no history of previous drug exposure, and the rates of previous exposure to hormones, 5‐ASA, and other drugs (including immunosuppressive agents) were 4.3%, 2.2%, and 39.1%, respectively (Table 1).

**TABLE 1:** Demographic characteristics.

Variables	
Number of patients	46
Age, years (IQR)	13.42 (11–14.44)
Sex, *n* (%)	
Female	18 (39.1)
Male	28 (60.9)
Height, cm (mean ± SD)	149.54 ± 14.64
Weight, kg (mean ± SD)	33.39 ± 9.08
BMI (IQR), *n* (%)	14.6 (13.3–15.43)
Malnutrition (%) <P10	33 (71.7)
Moderate nutritional status (%) P10–P75	13 (28.3)
Superior nutritional status (%) P75–P97	0 (0)
Overweight to obese (%) >P97	0 (0)
Time of hospitalization, *n* (%)	
1	31 (67.4)
2	7 (15.2)
3	8 (17.4)
History of exposure to biological agents, *n* (%)	
Yes	0 (0)
No	46 (100)
Drug exposure, *n* (%)	
No	25 (54.3)
Hormone	2 (4.3)
5‐ASA	1 (2.2)
Others (IMM, etc.)	18 (39.1)

Abbreviations: 5‐ASA, 5‐aminosalicylic acid; BMI, body mass index; IMM, immunosuppressant; IQR, interquartile range; kg, kilogram; SD, standard deviation.

Early onset disease with Paris classification A1a (<10 years old) and A1b (10–17 years old) accounted for 21.7% and 76.1% of patients, respectively. The disease extent was L1 in 6.5% of patients, L2 in 6.5%, L3 in 58.7%, L4b in 6.5%, L3 + L4 in 10.9%, and L3 + L4b in 10.9%. The disease behaviors of B1, B2, B3, and B2B3 accounted for 45.7%, 50%, 2.2%, and 2.2%, respectively. The growth retardation rate was 71.7%, and the perianal disease positivity rate was 34.8%. The PCDAI was 38.73 ± 15.47 before the first administration of IFX, 4.3% at the remission stage, 23.9% at the mild stage, 19.6% at the moderate stage, and 52.2% at the severe stage. The SES‐CD was 6.83 ± 2.29. Regarding the frequency of TDM monitoring, proactive TDM1 was 100%, proactive TDM2 was 54.35%, proactive TDM3 was 19.57%, and proactive TDM4 was 4.35%. In terms of biochemical indicators, blood routine indicators included red blood cell count (RBC; ×1012/L): 4.33 (3.89–4.72), hemoglobin levels (Hb; g/L): 99.48 ± 13.53, hematocrit (Hct; %): 32.52 ± 4.17, white blood cell count (WBC; ×109/L): 8.39 (7.28–10.56), neutrophil count (Neut#; ×109/L): 5.92 (4.7–7.71), lymphocyte count (Lymph#; ×109/L): 1.95 ± 0.61, platelet count (PLT; ×109/L): 490.39 ± 183.1, inflammatory index ESR (mm/hr): 48.51 ± 26.4, and CRP (mg/L): 29 (18.25–39.5). Liver and kidney function test results were as follows: total protein (TP; g/L): 67.58 ± 7.59, albumin (ALB; g/L): 35.9 ± 5.73, prealbumin (PA; mg/L): 30.9 (27.15–34.33), blood urea nitrogen (BUN; mmol/L): 2.85 (2.3–4.1), creatine (Cr; µmol/L): 43.47 ± 13.22, and uric acid (UA; µmol/L): 236.6 ± 86.11 (Table 2).

**TABLE 2:** Clinical characteristics at baseline.

Variables	
Diagnosis age, years, *n* (%)	
A1a 0–<10	10 (21.7)
A1b 10–<17	35 (76.1)
A2 17–40	1 (2.2)
A3 >40	0 (0)
Disease extent, *n* (%)	
L1	3 (6.5)
L2	3 (6.5)
L3	27 (58.7)
L4a	0 (0)
L4b	3 (6.5)
L3 + L4	5 (10.9)
L3 + L4b	5 (10.9)
Behavior, *n* (%)	
B1	21 (45.6)
B2	23 (50)
B3	1 (2.2)
B2B3	1 (2.2)
Growth, *n* (%)	
G0	13 (28.3)
G1	33 (71.7)
Perianal lesions, *n* (%)	
No	30 (65.2)
Yes	16 (34.8)
PCDAI (mean ± SD)	38.73 ± 15.47
Remission, *n* (%)	2 (4.3)
Mild, *n* (%)	11 (23.9)
Moderate, *n* (%)	9 (19.6)
Severe, *n* (%)	24 (52.2)
SES‐CD (mean ± SD)	6.83 ± 2.29
Proactive TDM, *n* (%)	46 (100)
TDM frequency	
TDM1, *n* (%)	46 (100)
TDM2, *n* (%)	25 (54.35)
TDM3, *n* (%)	9 (19.57)
TDM4, *n* (%)	2 (4.35)
Biochemical index	
RBC (IQR)	4.33 (3.89–4.72)
Hb (mean ± SD)	99.48 ± 13.53
Hct (mean ± SD)	32.52 ± 4.17
WBC (IQR)	8.39 (7.28–10.56)
ANC (IQR)	5.92 (4.7–7.71)
LNC (mean ± SD)	1.95 ± 0.61
PLT (mean ± SD)	490.39 ± 183.1
CRP (IQR)	29 (18.25–39.5)
ESR (mean ± SD)	48.51 ± 26.4
TP (mean ± SD)	67.58 ± 7.59
ALB (mean ± SD)	35.9 ± 5.73
PA (IQR)	30.9 (27.15–34.33)
BUN (IQR)	2.85 (2.3–4.1)
Cr (mean ± SD)	43.47 ± 13.22
UA (mean ± SD)	236.6 ± 86.11

Abbreviations: ALB, albumin; ANC, absolute neutrophil count; BUN, blood urea nitrogen; Cr, creatine; CRP, C‐reactive protein; ESR, erythrocyte sedimentation rate; Hb, hemoglobin; Hct, hematocrit; IQR, interquartile range; LNC, absolute lymphocyte count; PA, prealbumin; PCDAI, pediatric Crohn’s disease activity index; PLT, platelets; SD, standard deviation; SES‐CD, simplified endoscopic score for Crohn’s disease; TDM, therapeutic drug monitoring; TP, total protein; UA, uric acid; WBC, white blood cell.

### 3.2 Proactive trough concentration monitoring analysis

In total, 46 patients with initial TDM results were included in the analysis. According to the trough concentration (TC) (IQR), they were divided into insufficient (1 [0.4–18], *n* = 22), efficient (4.15 [3.8–4.95], *n* = 14), and sufficient (11.5 [9.5–19.775], *n* = 10) groups. The difference was statistically significant (*p* < 0.001). Anti‐infliximab antibody (ATI) analysis showed that four cases were ATI‐positive, all in the insufficient concentration group (ATI‐negative rate 81.8%); the ATI‐negative rate in the efficient and sufficient groups was 100%. At TDM1 monitoring, the IFX usage time (IQR) was 4.5 (4–9.25), 4.5 (4–10), and 4 (3.75–5), *p* = 0.235 in the insufficient, efficient, and sufficient groups, respectively. The PCDAI scores were 5 (4.38–13.13), 10 (5–12.5), and 5 (0–7.5) in the insufficient, efficient, and sufficient groups, respectively (*p* = 0.116). According to the PCDAI score, the proportions of the three groups achieving remission were 59.1%, 50%, and 80%; mild activity was 50%, 36.4%, and 20%, respectively; and severe activity was 4.5% in the insufficient group. Twenty‐one patients completed endoscopic evaluation at TDM1, including 10 patients in the insufficient group (SES‐CD score 4 [1–7]), 8 patients in the efficient group (3 [0–3]), and 3 patients in the sufficient group (6 [3–8]) *p* = 0.181); biochemical response rate (80%), CR rate (80%), and CRe rate (100%) were the highest in the sufficient group. The ERe rates were 50%, 87.5%, and 33.3% in the three groups. The mucosal healing rate was 37.5% in the efficient group (Table S1).

To identify the baseline factors affecting the TLI at TDM1, patients were divided based on IFX TC into sufficient (including efficient group) and insufficient level groups. Baseline data, including age, sex, weight, height, BMI, hormone exposure, SES‐CD, PCDAI, disease diagnosis data, perianal lesions, and biochemical parameters, were analyzed using a univariate analysis. SES‐CD (*p* = 0.014), perianal lesions (*p* = 0.043), TP (*p* = 0.048), BUN (*p* = 0.047), and PA (*p* = 0.07) were correlated with TLI levels. Furthermore, by including indicators with *p* < 0.15 in the univariate analysis, the multivariate analysis showed that hormone exposure (odds ratio [OR] = 0.042, 95% confidence interval [CI]: 0.002–0.924, *p* = 0.044), SES‐CD (OR = 1.656, 95% CI: 1.065–2.577, *p* = 0.025), perianal lesions (OR = 5.813, 95% CI: 0.984–34.349, *p* = 0.052), and TP (OR = 0.851, 95% CI: 0.749–0.967, *p* = 0.014) were correlated with TC levels (Table 3).

**TABLE 3:** Analysis of baseline factors associated with IFX trough levels at first proactive treatment drug monitoring.

Parameter	Univariate analysis OR (95% CI)	*p*‐value	Multivariate analysis OR (95% CI)	*p*‐value
Hormone exposure	0.181 (0.019–1.691)	0.134	0.042 (0.002–0.924)	**0.044**
SES‐CD	1.481 (1.082–2.026)	**0.014**	1.656 (1.065–2.577)	**0.025**
Age, years	0.836 (0.655–1.067)	0.151		
Behavior, *n* (%)	0.879 (0.356–2.17)	0.78		
Perianal lesions	3.8 (1.044–13.83)	**0.043**	5.813 (0.984–34.349)	0.052
Sex (male)	1.25 (0.381–4.104)	0.713		
Growth (G0)	1.098 (0.303–3.975)	0.887		
Height (m)	0.99 (0.951–1.031)	0.639		
Weight (kg)	0.994 (0.932–1.06)	0.859		
BMI (malnutrition)	0.911 (0.252–3.297)	0.887		
PCDAI	0.998 (0.961–1.037)	0.924		
Biochemical index				
TP	0.913 (0.835–0.999)	**0.048**	0.851 (0.749–0.967)	**0.014**
BUN	0.557 (0.312–0.992)	**0.047**	0.412 (0.153–1.114)	0.081
PA	0.903 (0.809–1.008)	**0.07**		
RBC	1.164 (0.609–2.225)	0.646		
Hb	1.026 (0.981–1.073)	0.262		
Hct	1.095 (0.945–1.268)	0.228		
WBC	1.061 (0.915–1.229)	0.433		
ANC	1.062 (0.905–1.246)	0.46		
LNC	1.795 (0.657–4.905)	0.254		
PLT	1 (0.997–1.003)	0.919		
CRP	0.978 (0.943–1.014)	0.222		
ESR	0.995 (0.976–1.015)	0.649		
ALB	0.961 (0.867–1.066)	0.457		
Cr	0.976 (0.932–1.023)	0.31		
UA	1 (0.994–1.007)	0.889		

Abbreviations: ALB, albumin; ANC, absolute neutrophil count; BMI, body mass index; BUN, blood urea nitrogen; CI, confidence interval; Cr, creatine; CRP, C‐reactive protein; ESR, erythrocyte sedimentation rate; Hb, hemoglobin; Hct, hematocrit; kg, kilogram; LNC, absolute lymphocyte count; OR, odds ratio; PA, prealbumin; PCDAI, pediatric Crohn’s disease activity index; PLT, platelets; RBC, red blood cell; SES‐CD, simplified endoscopic score for Crohn’s disease; TP, Total protein; UA, uric acid; WBC, white blood cell.

### 3.3 Analysis of proactive TDM‐guided treatment optimization

Of the 22 cases with insufficient TC by TDM1 monitoring, 16 cases (72.7%) had their infliximab treatment adjusted. 12 cases (54.5%) had their treatment interval shortened, 1 case (4.54%) had shortened treatment interval + increased dose, 1 case (4.54%) had shortened treatment interval + azathioprine, and 2 cases (9.1%) were switched to adalimumab. Two (9.1%) patients were lost to follow‐up, two (9.1%) did not continue on standard treatment, and two (9.1%) did not accept treatment adjustment (Table 4).

**TABLE 4:** First proactive treatment drug monitoring guiding treatment optimization.

IFX concentration, IQR/*n*	1 (0.4–18)/22
Insufficiency, *n* (%)	22 (100%)
ATI at TDM1, *n* (%)	
+	2 (9.1%)
‐	20 (90.9%)
Treatment adjustment after TDM1, *n* (%)	16 (72.7%)
Shorten the period, *n* (%)	12 (54.5%)
4w	6 (27.3%)
5w	3 (13.6%)
6w	3 (13.6%)
Shorten interval + increase dose	1 (4.5%)
Shorten interval + AZA	1 (4.5%)
Change to ADA	2 (9.1%)
Loss to follow‐up	2 (9.1%)
Non‐standard use	2 (9.1%)
No change	2 (9.1%)
With TDM2, *n* (%)	15 (68.2%)
With TDM3, *n* (%)	8 (36.4%)
With TMD4, *n* (%)	2 (9.09%)

Abbreviations: ADA, adalimumab; ATI, antibodies to infliximab; AZA, azathioprine; IFX, infliximab; IQR, interquartile range; TDM1, first therapeutic drug monitoring; TDM2, second therapeutic drug monitoring; TDM3, third therapeutic drug monitoring; TDM4, fourth therapeutic drug monitoring; w, week.

**FIGURE 2:**
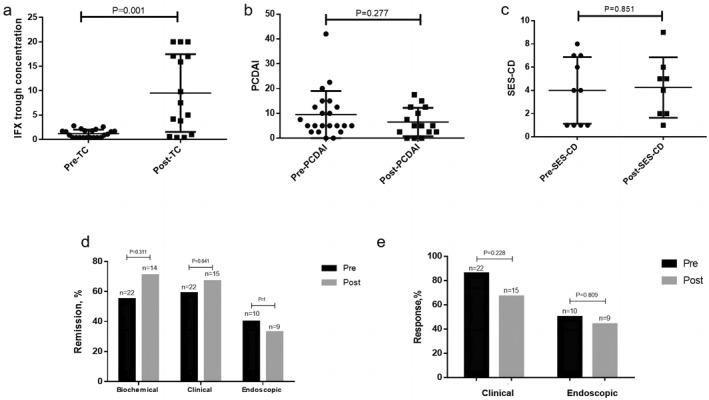
Effects of therapeutic adjustment on clinical outcomes. (A) IFX concentration before and after treatment adjustment. (B) PCDAI results before and after treatment adjustment. (C) SES‐CD before and after treatment adjustment. (D) Biochemical remission, clinical remission, and endoscopic remission rates before and after treatment adjustment. (E) Clinical response rate, and endoscopic response rate before and after treatment adjustment. PCDAI, pediatric Crohn's disease activity index; SES‐CD, simplified endoscopic scoring for Crohn's disease; TC, trough concentration.

The 22 patients were followed up, and proactive TDM monitoring showed that 15 patients had TDM2 (68.2%), 8 patients had TDM3 (36.4%), and 2 patients had TDM4 (9.09%). After treatment adjustment under the guidance of TDM1, concentration increased from 1 (0.4–18) at TDM1 to 6.05 (1.15–15) at TDM2 but decreased to 3.55 (0.4–12.075) at TDM3 and 3.75 (∼2.5) at TDM4 (Figure S1). TDM1 and TDM2 were used as evaluation time points before and after the treatment change. The interval between TDM1 and TDM2 was 93.9 ± 37.47 days. After the treatment change, the concentration increased (*p* = 0.001), PCDAI (*p* = 0.277) decreased, biochemical remission (*p* = 0.311), CR (*p* = 0.641), and ER rates increased (*p* = 1); SES‐CD (*p* = 0.851), CRe rate (*p* = 0.228), and ERe rate (*p* = 0.809) did not show a changing trend (Figure 2).

**FIGURE 3:**
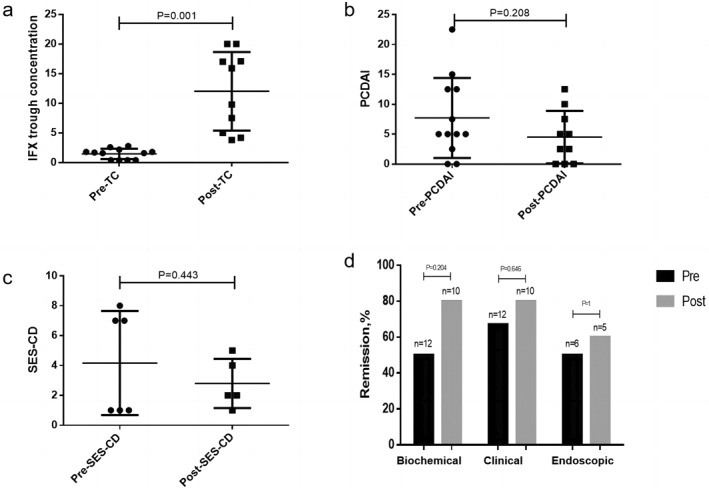
Effects of shortening treatment interval on clinical outcome. (A) IFX concentration before and after shortening treatment interval. (B) PCDAI before and after shortening treatment interval. (C) SES‐CD before and after shortening treatment interval. (D) Biochemical remission, clinical remission, and endoscopic remission rates before and after shortening treatment interval. PCDAI, pediatric Crohn's disease activity index; SES‐CD, simplified endoscopic scoring for Crohn's disease; TC, trough concentration.

Twelve patients with shortened treatment intervals were analyzed, including 10 who underwent TDM2 concentration monitoring. Shortening the interval increased the TC (1.69 ± 0.8 vs. 12.03 ± 6.64, *p* < 0.001) and decreased the PCDAI (6.5 ± 5.3 vs. 4.5 ± 4.38, *p* = 0.208) and SES‐CD (4.5 ± 4.95 vs. 1.5 ± 0.71, *p* = 0.443). The rates of biochemical remission (50% vs. 80%, *p* = 0.20), CR (66.7% vs. 80%, *p* = 0.65), ER (50% vs. 60%, *p* = 1), and ERe (50% vs. 80%, *p* = 0.54) improved (Figure 3).

## 4 DISCUSSION

An analysis of the demographic characteristics and clinical indicators revealed that the overall nutritional status of the included patients was poor and that disease activity status was mainly in a state of severe activity at the time of the first IFX treatment.

TDM has emerged as one of the most sought‐after objective tools for assessing the therapeutic efficacy of biologics (in particular, anti‐TNF‐α) and immunomodulators in IBD.^34^, ^35^ Herein, we enrolled patients with proactive TDM; an analysis of the factors that influence IFX TC indicated that hormone exposure, SES‐CD, perianal lesions, and TP levels during the first IFX use affected TC levels. In the optimization of proactive TDM‐driven treatment changes, shortening the treatment interval is a common choice for pediatricians, which can improve drug serum levels, reduce PCDAI, and improve the treatment efficacy. However, the maintenance effect of concentration may not be good. The sample size and follow‐up time should be further expanded to evaluate long‐term effects.

Proactive TDM utilizes the regular measurement of drug trough concentrations and ADAs with dose adaptation to target an appropriate drug TC.^36^ Preliminary research data found that proactive TDM of IFX can provide safer and more objective dose adjustments than empirical dose optimization or reactive TDM.^37^, ^38^ However, a lack of consistency in evidence still challenges proactive TDM in managing IBD therapy. When and how often monitoring should be used to reflect the role of the guiding function in treatment adjustment is under‐investigated.

We enrolled patients with pCD treated with IFX under proactive TDM. The relationship between IFX TC in TDM monitoring and the clinical treatment effect was analyzed according to the matched clinical data of each TDM. Patients with sufficient TCs had the highest biochemical remission, CR, and CRe rates at TDM1. Although this difference was not statistically significant, a trend was observed. Many studies have reported an association between serum IFX levels and clinical outcomes in IBD.^28^, ^39^, ^40^ A systematic review and meta‐analysis including 22 studies showed that a trough threshold >2 μg/mL during maintenance is associated with a greater probability of CR and mucosal healing.^41^ Children with clinical or ER had significantly higher IFX exposure during maintenance therapy.^27^

Furthermore, we analyzed which baseline factors influence TC to guide clinical practice. Univariate and multivariate analyses indicated that hormone exposure, SES‐CD, perianal lesions, and TP at baseline before the first use of IFX were correlated with IFX TC level at TDM1; the difference was statistically significant. Similar results have been reported in other research groups. A repeated‐measures study including adult patients with IBD revealed associations between ATI, serum albumin concentration, concomitant immunosuppressive therapy, body weight, sex, and IFX TC.^42^ BMI, concomitant use of immunomodulators, rates of side effects, and laboratory markers, including serum albumin and CRP, are significantly associated with anti‐TNF‐α trough levels in IBD patients.^43^

Twenty‐two cases had insufficient TC by TDM1 monitoring, among which 16 (72.7%) had treatment adjustment, including 12 (54.5%) cases of shortened treatment interval. The impact of TDM on clinical decision‐making for children receiving IFX for IBD is a meaningful topic. A previous study on IBD in children reported that TDM‐based IFX optimization improved clinical outcomes and that shortening dosage interval resulted in better IFX concentration than dose optimization.^44^ Deora et al. also found that TDM‐based treatment changes resulted in a significant clinical improvement.^45^ However, there are relatively few reports on proactive TDM‐guided treatment adjustment and dynamic TDM evaluation. In this study, pediatricians were more inclined to choose the shortened interval regimen when the concentration was insufficient. In this study, we also used several other program adjustments, such as shortening the treatment interval + increasing the dose, shortening the treatment interval + azathioprine, and switching biological agents to ADA. Due to the small sample size, we could not compare different protocols; however, these protocol adjustments might better improve the concentration. Switching to ADA was effective in the follow‐up of both cases. Dynamic TDM was performed in 22 patients, with 15 receiving TDM2, 8 receiving TDM3, and 2 receiving TDM4. By analyzing the concentration dynamics, we found that TDM2 concentration increased (6.05 [1.15–15]); however, TDM3 (3.55 [0.4–12.075]) and TDM4 (3.75 [∼2.5]) concentrations decreased, indicating that the increased concentration may not be maintained in the long term. Furthermore, 12 patients with TDM‐guided shortened intervals were analyzed, and 10 had TDM2 (the interval between TDM1 and TDM2 was about 90 days). Clinical evaluation before and after interval shortening suggested that the adjustment of the interval shortening protocol could improve the concentration level, reduce PCDAI and SES‐CD, and improve the biochemical response, CRe, and ERe rates after 3 months. However, the sample size needs to be expanded for further evaluation. To our knowledge, clinical studies on optimal IFX treatment guided by TDM for children with CD are limited. Lega et al. reported that proactive TDM might improve IFX durability by maintaining higher IFX concentrations during maintenance.^46^ A review of IFX use in adult patients with perianal fistulizing CD indicated that the optimal timing of IFX use was highly individualized.^47^ Therefore, further clinical research on scheme optimization based on TDM guidance is of clinical value.

The strengths of this research include proactive TDM‐based treatment optimization, dynamic, proactive TDM monitoring, and pre‐and post‐treatment effect evaluation. The small sample size and retrospective design are the major limitations of this study. Moreover, inter‐group comparison between different optimization protocols cannot be conducted. Additionally, data retrieval under the set inclusion criteria does not reflect the real situation of all patients. Further, short follow‐up time and lack of a control cohort are other limitations. Future investigations with a larger sample size and long‐term follow‐up are warranted. Further prospective large‐sample clinical studies implementing natural outcome control, superposition of different optimization schemes, and switching biological drugs, would provide a more comprehensive approach and better guidance for clinical practice.

## 5 CONCLUSION

Hormone exposure, SES‐CD, perianal lesions, and TP levels during the first IFX use were factors that affected TC. The shortened interval adjustment guided by proactive TDM can improve IFX concentration and biochemical remission, CR, and ER rates after 3 months. However, the long‐term maintenance effect of the concentration may require further study, long‐term follow‐up, and evaluation of health and economic value.

## AUTHOR CONTRIBUTIONS

The authors confirm contribution to the paper as follows: study conception and design: Xiaoqin Zhou, Jinlin Song; data collection: Junya Song, Huihui Zhang, Huihua Zhang, Xue Zhan, Xiaomei Song, Hong Guo; interpretation of results: Junya Song, Huihui Zhang, Xiaoqin Zhou; manuscript preparation: Junya Song, Huihui Zhang, Xiaoqin Zhou, Huihua Zhang, Ximing Xu, Xiaohua Liang, Yongfang Liu, Xue Zhan. All authors contributed to the article and approved the submitted version.

## CONFLICT OF INTEREST STATEMENT

The authors declare that the research was conducted in the absence of any commercial or financial relationships that could be construed as a potential conflict of interest.

## ETHICS STATEMENT

This study was approved by the Ethics Committee of the Children's Hospital of Chongqing Medical University and Chongqing General Hospital (approval no. 2023 (169), KY S2022‐023‐01). Written informed consent was obtained from all patients and their parents.

## Supporting information

Supporting Information S1

## Data Availability

The original contributions presented in the study are included in the article/supplementary material; further inquiries can be directed to the corresponding authors.
