# Supplementary material for: The role of proactive therapeutic drug monitoring in guiding infliximab therapeutic optimization in pediatric patients with Crohn's disease: A retrospective study
Source: Pediatr Discov. 2024 Jun 25;2(4):e96. doi: 10.1002/pdi3.96 (PMC12118267; doi:10.1002/pdi3.96)
Supplement: Supplementary file 1 — Supporting Information S1 [file PDI3-2-e96-s001.docx]

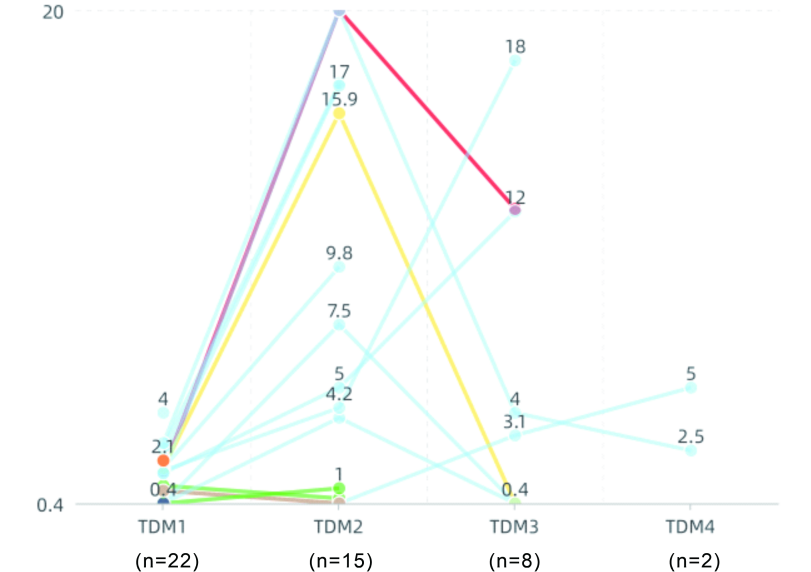


Figure S1. Dynamic monitoring of concentration changes after adjustment of the different therapeutic schedules

TDM, therapeutic drug monitoring.

Table S1. Analysis of first proactive treatment drug monitoring

| **Table S1 Analysis of first proactive treatment drug monitoring** | | | | |
| --- | --- | --- | --- | --- |
| Categories / Groups | Insufficient group  (n=22) | Efficient group (n=14) | Sufficient group  (n=10) | P |
| TC (IQR) | 1(0.4-18) | 4.15(3.8-4.95) | 11.5(9.5-19.775) | 0 |
| ATI |  |  |  |  |
| + | 4(18.2) | 0(0) | 0(0) | 0.126 |
| - | 18(81.8) | 14(100) | 10(100) |  |
| IFX usage times (IQR) | 4.5(4-9.25) | 4.5(4-10) | 4(3.75-5) | 0.235 |
| PCDAI (IQR) | 5(4.38~13.13) | 10(5~12.5) | 5(0~7.5) | 0.116 |
| Remission, n(%) | 13(59.1) | 7(50) | 8(80) | 0.494 |
| Mild, n(%) | 8(36.4) | 7(50) | 2(20) |  |
| Moderate, n(%) | 0(0) | 0(0) | 0(0) |  |
| Severe, n(%) | 1(4.5) | 0(0) | 0(0) |  |
| SES-CD, IQR/n | 4(1~7)/10 | 3(0~3)/8 | 6(3~8)/3 | 0.181 |
| Biochemical remission, n(%) | 11(55) | 8(57.1) | 8(80) | 0.442 |
| Clinical remission, n(%) | 13(59.1) | 7(50) | 8(80) | 0.388 |
| Clinical response, n(%) | 21(95.5) | 14(100) | 10(100) | 1 |
| Endoscopic remission, n(%) | 4(40) | 3(37.5) | 0(0) | 0.675 |
| Endoscopic response, n(%) | 5(50) | 7(87.5) | 1(33.3) | 0.155 |
| Mucosal healing, n(%) | 0(0) | 3(37.5) | 0(0) | 0.083 |

TC, trough concentration;IQR,interquartile range;ATI,An antibody to infliximab; IFX, infliximab;

PCDAI,pediatric Crohn’s disease activity index;SES-CD,simplified endoscopic score for Crohn’s disease.
